# Chromatin complex dependencies reveal targeting opportunities in leukemia

**DOI:** 10.1038/s41467-023-36150-7

**Published:** 2023-01-27

**Authors:** Fadi J. Najm, Peter DeWeirdt, Molly M. Moore, Samantha M. Bevill, Chadi A. El Farran, Kevin A. Macias, Mudra Hegde, Amanda L. Waterbury, Brian B. Liau, Peter van Galen, John G. Doench, Bradley E. Bernstein

**Affiliations:** 1 Gene Regulation Observatory, Broad Institute of MIT and Harvard, Cambridge, MA USA; 2 Genetic Perturbation Platform, Broad Institute of MIT and Harvard, Cambridge, MA USA; 3 Department of Cancer Biology, Dana-Farber Cancer Institute, Boston, MA USA; 4 Departments of Cell Biology and Pathology, Harvard Medical School, Boston, MA USA; 5 Department of Chemistry and Chemical Biology, Harvard University, Cambridge, MA USA; 6 Division of Hematology, Brigham and Women’s Hospital, Boston, MA USA

**Keywords:** Functional genomics, Cancer genomics, Leukaemia

## Abstract

Chromatin regulators are frequently mutated in human cancer and are attractive drug targets. They include diverse proteins that share functional domains and assemble into related multi-subunit complexes. To investigate functional relationships among these regulators, here we apply combinatorial CRISPR knockouts (KOs) to test over 35,000 gene-gene pairings in leukemia cells, using a library of over 300,000 constructs. Top pairs that demonstrate either compensatory non-lethal interactions or synergistic lethality enrich for paralogs and targets that occupy the same protein complex. The screen highlights protein complex dependencies not apparent in single KO screens, for example MCM histone exchange, the nucleosome remodeling and deacetylase (NuRD) complex, and HBO1 (KAT7) complex. We explore two approaches to NuRD complex inactivation. Paralog and non-paralog combinations of the KAT7 complex emerge as synergistic lethal and specifically nominate the ING5 PHD domain as a potential therapeutic target when paired with other KAT7 complex member losses. These findings highlight the power of combinatorial screening to provide mechanistic insight and identify therapeutic targets within redundant networks.

## Abstract (web summary)

Epigenetic regulators are potential therapeutic drug targets in leukemia. Here, the authors perform combinatorial CRISPR knockouts to test gene-gene pairings in leukemia cells to discover compensatory non-lethal or synergistic lethal combinations with therapeutic potential.

## Introduction

Gene expression is modulated by dynamic chromatin states controlled by diverse families of chromatin regulators. These regulators are widely expressed across tissues and cooperate with a host of other factors to direct and stabilize cell fate decisions^1–4^. Chromatin aberrations have been implicated in many diseases, including cancer^5–7^. While this supports a potentially broad therapeutic opportunity, the redundancy and complex interplay between chromatin regulators has constrained the identification of targets and indications. Recent combinatorial CRISPR based systems have enabled the discovery of novel pairwise dependencies^8–16^. Paralogs, homologous genes sharing sequence similarity and overlapping function, surfaced as high yield targets. This has stimulated the field to focus on screening paralog combinations. Therefore, a challenge remains as to which non-paralog pairs to explore and in which contexts they function.

In this work, we apply pairwise CRISPR tools to screen chromatin regulator combinations for cancer dependencies that may present therapeutic opportunities. We identify paralog and non-paralog dependencies and investigate dependent gene pairs whose protein products associate in transcriptional complexes. We discover a dependency in a paralog pair due to low expression of a third paralog partner in the cell lines tested. We find that non-paralog partners found in the same protein complex sometimes score as hits. Our combinatorial screening results provide a basis for further functional exploration including the development of therapeutics for leukemia.

## Results

### Pairwise combinatorial chromatin regulator screening

We selected 268 chromatin regulators that contain one or more of 50 protein family (Pfam) domains with the rationale that the domains could ultimately enable target prosecution (Supplementary Data 1 and 2, Supplementary Fig. 1a). We initially produced a pilot library targeting 98 chromatin regulator genes and screen pairwise combinations all-by-all in THP-1, a mixed-lineage leukemia (MLL)-AF9 rearranged monocytic leukemia, and Reh, a TEL-AML1 carrying lymphocytic leukemia (Supplementary Fig. 2a-c). We leveraged our CRISPR system that achieves pairwise gene inactivation by delivering two guide RNAs (sgRNAs), one of which directs *S. pyogenes* Cas9 and a second that directs *S. aureus* Cas9^8^. We identified several gene pairs that were significantly depleted relative to either gene alone in both lines, including *HDAC1;HDAC2* and *BRD2;BRD3* (Supplementary Fig. 3a–d). Prior work has shown *HDAC1;HDAC2* as a synergistic pair in many tissues^11,12^. The pilot screen confirmed our ability to screen and identify significant chromatin regulator pairs.

**Fig. 1:**
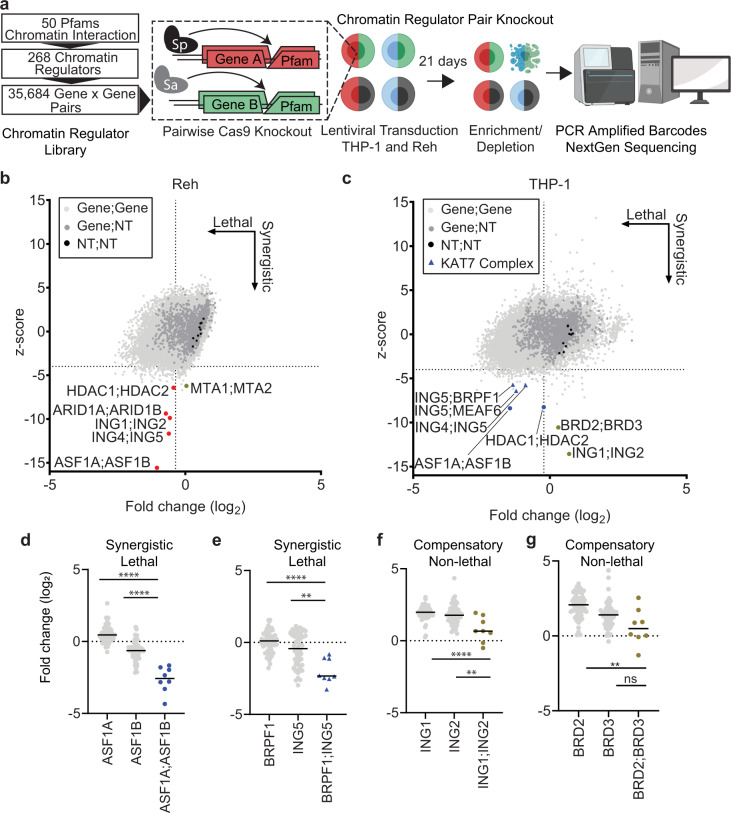
Systematic screening of chromatin regulator pairs. **a** Combinatorial screening workflow with *S. pyogenes* (Sp) and *S. aureus* (Sa) Cas9s (generated using BioRender). **b**, **c** Knockout data from the 300k library screen (*n* = 35,684 gene pairs) with non-targeting combinations (black circles), single knockouts (dark gray circles), depleting combinations labeled for Reh in **b** (red circles) and THP-1 in **c** (blue circles), KAT7 complex members (blue triangles), and compensatory non-lethal pairs (olive circles). Dotted lines at z-score of −4 and average log2 fold change, −0.36 for Reh and −0.27 for THP-1. Data are from duplicate screens. Screening data available in Supplementary Data 3. **d**–**g** Primary screening data for a gene-gene combination (*n* = 8 sgRNA pairs) or gene-non-targeting combination (*n* = 56 sgRNA pairs) in THP-1 for: ASF1A and ASF1B in **d**, KAT7 complex members BRPF1 and ING5 in **e** (**P = 0.002), ING1 and ING2 in **f** (**P = 0.0059), and BRD2 and BRD3 in **g** (**P = 0.0015). Non-lethal pairings are highlighted (olive circles). Solid black line indicates mean. Data are from duplicates with example replicate shown. *P*-values are based on the two-tailed Mann–Whitney test, **P < 0.01; ***P < 0.001; ****P < 0.0001. Source data are provided as a Source Data file.

We next scaled our approach by generating a full library of the 268 genes, designing 4 sgRNAs per gene targeted to the chromatin interacting Pfam domains^17^. We included non-targeting and essential gene targeting control sgRNAs for a total library size of 304,704 constructs (300k library) (Fig. 1a, Methods). Log_2_ fold changes (LFCs) were calculated for each construct and used to determine lethality. Average LFC was −0.36 for Reh and −0.27 for THP-1 (Fig. 1b, c). Correlation of log-fold changes between Cas9 orthologs (Pearson coefficients 0.81–0.85) and experimental replicates in each cell line (Pearson coefficients 0.93–0.95) demonstrated strong technical performance and in line with prior efficiencies (Supplementary Fig. 4a, b)^8^. Single KO essential genes correlated well to previously published screens^18^ (Pearson coefficients 0.80 and 0.63) and we further binned these genes based on cell line and a core essential gene list^19^ (Supplementary Fig. 1b, 5a–d). To measure genetic interactions, for each sgRNA we fit a line between the LFCs of the sgRNA in combination with other targeting sgRNAs versus the LFCs of each sgRNA paired with negative controls. We then z-scored the residuals from these fit lines, and combined scores across all sgRNAs targeting the same gene pair^20^. We defined synergistic combinations as those with z-score less than −4, while lethality was measured based on an LFC less than the average.

Gene paralogs result from evolutionary duplication events and often share overlapping functions. Prior studies have shown that paralogs are less likely than singleton genes to score as essentials in yeast and human whole genome screens^12,14,21–23^. We define paralog genes as those with greater than 25% sequence similarity. By this definition, 75% of these chromatin regulators have a paralog compared to 67–71% for the whole genome (Supplementary Fig. 1c)^24^. Numerous paralog pairs, including the *ASF1A;ASF1B* combination, scored in our screen as synergistic lethal in both Reh and THP-1 (Fig. 1b–d). ASF1A and ASF1B are H3-H4 histone chaperones with 70.5% sequence similarity and play critical roles in histone exchange^25^. Their combination was also identified in a previous screen^14^, and likely represents a core synthetic lethal pair across cell types. Synergistic scoring of paralogs responsible for critical cellular functions strengthened our confidence in screening results.

We observed substantial differences between the two screened cell models. In Reh, the top performing synergistic lethal hits were primarily paralogs, representing 6 of the top 10 z-score combinations (Fig. 1b). In THP-1 we observed a paralog pair hit as well as synergistic lethal pairs of KAT7 protein complex members. Combinations involving ING5 paired with ING4, BRPF1, or MEAF6, were strongly depleted in THP-1 (Fig. 1c, e). While in Reh ING5 was only depleted with its paralog, ING4 (Fig. 1b). These data extend earlier observations of KAT7 dependencies in MLL-rearranged leukemia^26,27^. Results here highlight opportunities for synergy with KAT7 complex partners to be explored further (see below).

Functional compensation, where loss of a gene is moderated by a related gene, was also detected between certain pairs in THP-1. Positive LFC trends across z-scores were observed in THP-1 more than in Reh (Fig. 1b, c). Among the top z-score depleted were *ING1;ING2* and *BRD2;BRD3*. Single KO of *ING1, ING2, BRD2* and *BRD3* in THP-1 led to increased LFCs of 1.8, 1.4, 1.3, and 0.9 respectively, consistent with improved fitness upon gene loss (Fig. 1f, g, Supplementary Figs. 5 and 6, Supplementary Data 3). However, combinatorial *ING1;ING2* or *BRD2;BRD3* KOs suppressed the effects (LFC near 0), though the double KOs remained viable (Fig. 1f, g). Therefore, we labeled these combinations as compensatory. In sum, compensation in THP-1 was distinct from synergistic lethal events and categorized as compensatory non-lethal.

### Validation screening in additional leukemia models

**Fig. 2:**
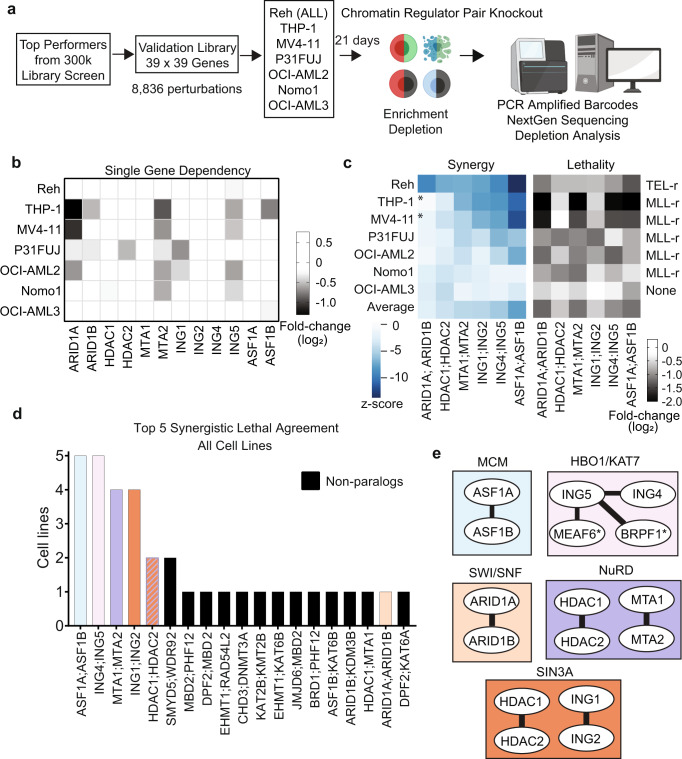
Synergistic pairs converge on protein complexes and redundant paralogs. **a** Validation screen workflow in 7 leukemia cell lines (generated using BioRender). ALL, acute lymphocytic leukemia. **b** Single knockout for top performing gene pairs tested in validation library. Data are the mean of duplicate screens. **c** z-score and log fold-change heatmaps of paralog combinations meeting a 1 × 10^−5^ FDR threshold. Rearrangements denoted with an “r” for TEL-r or MLL-r carrying cell lines. THP-1 and MV4-11 are susceptible to ARID1A knockout alone, denoted with asterisk (*). Data are derived from duplicates. **d** Bar plot depicting overlap of the top 5 synergistic lethal hits from each of the 7 indicated cell lines as determined by LFC and z-score. Paralog pairs highlighted by complex color denoted in **e** and non-paralogs in black. **e** Top scoring gene pairs from the 300k library screen organized by protein complexes they comprise. Asterisk (*) indicates MEAF and BRPF1 were not tested in the validation screen. Source data are provided as a Source Data file.

To evaluate the generality of synergistic lethal pairs, we screened 5 additional leukemia cell lines with varying MLL rearrangement status (see Methods). We chose 39 synergistic genes from the 300k library screen, designed a validation library totaling 8,836 constructs (2 sgRNAs per gene for each Cas9 ortholog, paired all-by-all), and screened in the 7 leukemia cell lines (Fig. 2a, b, Supplementary Data 4). THP-1 and Reh LFC results in this validation screen were highly concordant with the 300k library screen (Pearson coefficients of 0.77 and 0.84), and replicate correlation for all cell lines were high (Pearson coefficient range of 0.82 to 0.95) (Supplementary Fig. 7a–c). The highest scoring synergistic lethal pairs across most of the cell lines were paralog genes (Fig. 2c, d). In these instances, genetic redundancy protected the critical functions of histone deposition (*ASF1A;ASF1B*), transcriptional activation (*ING4;ING5*), and transcriptional repression (*MTA1;MTA2, ING1;ING2 and HDAC1;HDAC2*). Some of these paralog combinations may be universally lethal and promising therapeutic targets when a partner is inactivated in cancer.

We next related synergistic combinations to corresponding chromatin regulator complexes. Connectivity maps of the top synergistic lethal hits revealed several distinct complexes (Fig. 2e). Histone chaperones ASF1A and ASF1B multimerize within the minichromosome maintenance complex (Fig. 2c–e). Other members of the complex, including CHAF1A, CHAF1B and HIRA scored individually as essentials in DepMap for all lines tested, and thus would not be synergistic in combinations (Supplementary Fig. 8a, b). The SWI/SNF chromatin remodeling components *ARID1A:ARID1B* scored in Reh cells (Fig. 2c). ARID1A and ARID1B are 60% identical, mutually exclusive in SWI/SNF chromatin remodeling complexes, and an established synthetic lethal dependency in cancers^28,29^. Although *ARID1A;ARID1B* did not score in the AML cells, we observed a single KO dependency on *ARID1A* alone in THP-1 and MV4-11 (Fig. 2b, Supplementary Fig. 8c). Thus, single and pairwise KOs provided insight into redundant and non-redundant protein complex dependencies.

### Dependencies in transcriptional chromatin complexes

Complexes that modulate gene transcription play an important role in cancer progression. Redundancy among members of the SIN3A, NuRD, and KAT7 complexes were identified from combinatorial screening. The SIN3A repressive complex members *HDAC1;HDAC2* and *ING1;ING2* were synergistic hits in our validation screen (Fig. 2d, e and Supplementary Fig. 8d–f). Although not explored in depth here, SIN3A complex genetic interactions represent an opportunity for further investigation. We focused here on the NuRD and KAT7 complexes.

**Fig. 3:**
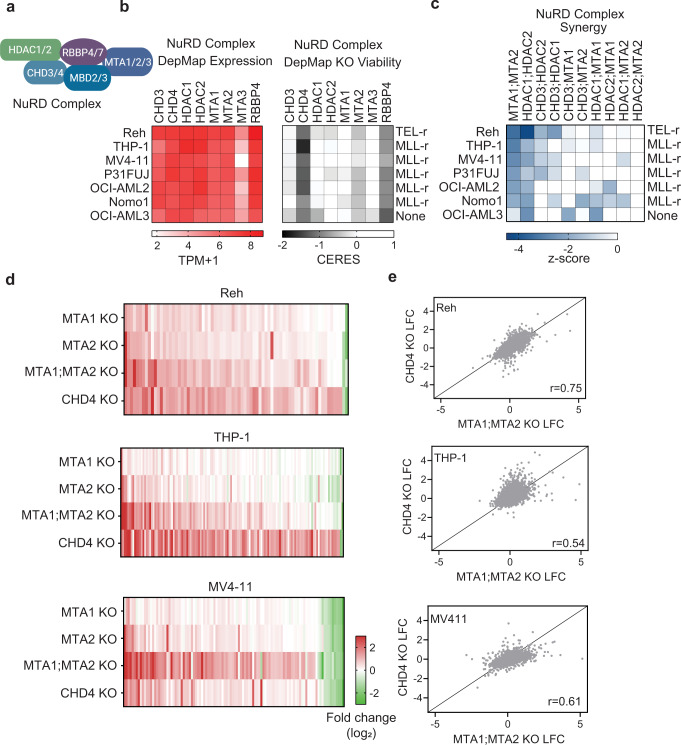
Two mechanisms for NuRD loss. **a** NuRD complex schematic. **b** Heatmaps of expression and single knockout data for select members of the NuRD complex. Rearrangements were denoted with an “r” for TEL-r or MLL-r carrying cell lines. DepMap 21q1 release. **c** Heatmap of combinatorial knockouts of top NuRD complex pairings tested in follow up screen. **d** Gene expression heatmaps of DEseq differential genes in indicated knockout conditions for 7 days in Reh (75 genes), THP-1 (132 genes) and MV4-11 (108 genes). Data are normalized to pairwise non-targeting sgRNA transduced cells and average of 2 replicates per condition, TPM + 1. Flow cytometry gating strategy found in Supplementary Fig. 10. **e** Scatter plot depicting log2-fold change expression in MTA1;MTA2 KO vs. CHD4 KO for THP-1, Reh and MV4-11. Pearson correlations denoted on each plot. RNAseq data provided in Supplementary Data 5. Source data are provided as a Source Data file.

The NuRD complex is a transcriptional repressor that combines the enzymatic activities of ATPase-dependent nucleosome remodelers and histone deacetylases to fine tune enhancer activity^30–32^. The complex has at least 5 components with several inter-changeable factors (Fig. 3a). Although CHD3 and CHD4 paralogs share 65% sequence similarity, only CHD4 is essential (Fig. 3b). Recently it was demonstrated that MTA1/2/3 triple KO in embryonic stem cells creates a non-lethal NuRD loss of function model^33^. In the leukemia cell lines tested, MTA3 is not expressed and MTA2 is a mild dependency (Fig. 2b, b). Strikingly, combined KO of MTA1 and MTA2 resulted in robust synergistic and lethal effects in most of the cell lines (Figs. 2c and 3c). The strongest pairwise lethality was observed in lines carrying MLL-r fusions. We postulated that KO of both MTA1 and MTA2 may confer NuRD loss of function and offer a cell-type specific opportunity.

We examined the transcriptional impact of *MTA1;MTA2* KO versus CHD4 KO as two potential mechanisms for NuRD loss. We cloned our validated sgRNAs into EGFP and mCherry reporter lentiviral vectors and transduced Reh, THP-1 and MV4-11 cell lines. After 7 days, we performed RNA-seq and identified differential genes in single and paired KO samples (Fig. 3d and Supplementary Data 5). *MTA1;MTA2* and CHD4 KOs each led to more upregulated genes, suggesting loss of the NuRD repressive complex. This data emphasized the importance of combinatorial targeting over single MTA protein KO due to rich gene expression changes with pairwise KO. Furthermore, single KOs of *MTA1, MTA2* or *CHD4* in Reh led to increased expression of the other genes (Supplementary Fig. 9a). This adaptive response may explain the weaker response to *MTA1;MTA2* KO in Reh. Expression patterns in *CHD4* KO and *MTA1;MTA2* KO were relatively similar within each cell line (correlations ranged 0.56–0.75) and much weaker between cell lines (0.09–0.28 for *CHD4* KO and 0.15–0.35 for *MTA1;MTA2* KO) (Fig. 3e and Supplementary Fig. 9b). We observed upregulation of a subset of macrophage differentiation genes in response to either *CHD4* or *MTA1;MTA2* KO, which was more pronounced in THP-1 (Supplementary Fig. 9c). Taken together, we find that the NuRD complex likely represses in a cell type specific manner and hence maintains an undifferentiated state and cell viability in specific contexts. These experiments present a dual approach for inactivating the NuRD complex that may be useful for biological discovery and therapeutics.

**Fig. 4:**
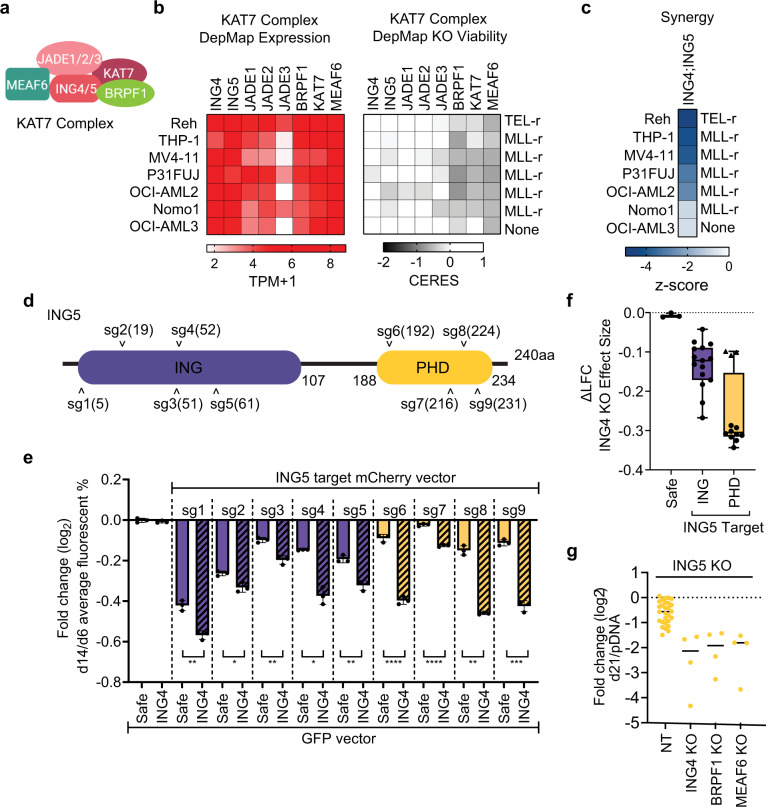
ING5 synergy with KAT7 complex partners. **a** KAT7 complex schematic. **b** Expression and single knockout heatmaps for select members of the KAT7 complex. DepMap 21q1 release. **c** Heatmap of combinatorial knockouts of top KAT7 complex pairings tested in validation screen. Data are repeated from Fig. 2c. **d** ING5 protein annotated with ING (purple) and PHD (yellow) Pfam domains and sgRNA targeting sites. **e** Competition experiment of ING5 knockout single sgRNAs delivered by mCherry vector and ING4 KO or safe harbor targeting (Safe) sgRNAs delivered by GFP vector to THP-1 cells and analyzed by flow cytometry after 6 and 14 days. Sample represents 3 replicates collected at each respective timepoint. Data are presented as mean values ± standard deviation. See Supplementary Fig. 10 for flow gating strategy and Supplementary Data 6 for values. *P*-values are based on the two-tailed Welch’s *t* test, *P < 0.05; **P < 0.01; ***P < 0.001; ****P < 0.0001 **f**, Log_2_ fold change differences for all tested ING5 ING and PHD targeting sgs in ING4 KO cells. Triangles represent sg7. Data presented as median, boxes denoting upper and lower quartiles and whiskers the range. **g** Relative read frequency of ING5 PHD knockout when paired with non-targeting (NT, 14 sgRNAs), ING4 (2 sgRNAs), BRPF1 (2 sgRNAs), BRPF1 (2 sgRNAs), and MEAF6 (2 sgRNAs) after 21 days in THP-1. Combinations were tested in duplicate, solid black bars at mean. Source data are provided as a Source Data file.

KAT7 acetyltransferase catalyzes histone H3 acetylation and regulates transcription and various other cell functions. KAT7 has been implicated as a dependency in MLL-rearranged leukemia with inhibition of enzymatic activity as the therapeutic focus^26,27,34^. The KAT7 complex consists of KAT7, MEAF6, ING4, or ING5, and either a JADE or BRPF member; most of which are expressed in leukemia lines used in this study (Fig. 4a, b)^35^. While *ING4* KO was well-tolerated, *ING4;ING5* loss was synergistic lethal in 5 of the 7 tested lines (Figs. 2c, d and 4c, e). ING5 contains two major Pfam domains: the C-terminal PHD zinc-finger domain of ING5 that binds H3K4me3 to direct the complex to activation targets, and an N-terminal ING domain that binds unmodified H3 tails^36^. We tested multiple sgRNAs targeting the ING and PHD domains of *ING5* in THP-1 cells (Fig. 4d). All *ING5* sgRNAs resulted in a stronger loss when paired with *ING4* KO (Fig. 4e, f, Supplementary Fig. 10 and Supplementary Data 6). *ING5* PHD domain perturbations were generally consistent, and all showed synergy with *ING4* loss. *ING5* PHD domain sgRNAs also induced stronger lethality in combination with other KAT7 complex members (*BRPF1, MEAF6*) (Fig. 4g). The identification of synergistic lethal interactions among KAT7 complex members may be relevant for the development of therapies targeting the complex, including ongoing efforts to target KAT7 histone acetyltransferase activity^26^.

## Discussion

Here we screened 268 chromatin regulators in pairwise combinations to identify synergistic dependencies in leukemia. We define combinatorial hits based on synergy and lethality. Synergistic pairs converge on a relatively small number of protein complexes with roles in histone deposition, transcriptional activation, and transcriptional repression. Synergistic lethal combinations often center on loss of one protein resulting in mild effects that exacerbate with the loss of another complex member. We explore gene combinations of the NuRD and KAT7 chromatin complexes, determining the interplay and dependencies of these partners.

Our results highlight the importance of redundancy screening, as single KOs may miss important protein complex dependencies. A subset of redundant paralogs found in the same complex are dependent across an entire class of cells. While these targets are likely lethal in all cells, a cancer specific mutation in one of these genes may confer an opportunity for specificity. For example, ASF1A is deleted in 10% of diffuse large B-cell lymphoma TCGA samples, making ASF1B a possible target.

Several experimental and biological factors may challenge the identification of therapeutically relevant gene pairs. Pairwise all-by-all whole genome screens are not currently feasible, so targeted gene lists must be selected to limit focus, such as paralog combinations^12^ or the chromatin regulators in this study. We screened libraries of varied pool sizes here, ranging from approximately 9k to 300k perturbations. While larger pooled libraries enable screening breadth, additional perturbations dilute the effect of each measurement. For example, MTA1;MTA2 KO in THP-1 was not a significant lethal pairing in the 300k library screen but scored in the validation screen. Finally, we focused on 7 AML cell lines to determine context-specific interactions, recognizing that future studies will need to assess lethality of combinations in normal cell lines and tissues for therapeutic window determination.

Our pairwise screening enabled a dual approach to NuRD loss of function modeling and pointed toward synergistic lethality in MLL-rearranged leukemia. We observed ING4 and ING5 dependency, with apparent emphasis on the PHD domain targeting for synergy. Our results complement recent observations that MLL fusions associate with the KAT7 complex at oncogenic promoters^37^. While acetyltransferase inhibitors are under development for MLL-rearranged leukemia, the clinical potential of these compounds is unclear^38^. Strategies that target ING5 PHD domain and KAT7 complex members should also be considered. Taken together, our work uncovers chromatin regulator dependencies in leukemia cell lines that may be exploitable as cancer-cell specific therapeutic targets.

## Methods

### Cell culture

AML cell lines were first transduced with pLX_311-Cas9 (Addgene 96924), selected with blasticidin (2 µg/ml), and maintained in RPMI 1640 with glutamax (Gibco) with 10% (v/v) fetal bovine serum (FBS, Peak Serum) and penicillin/streptomycin (Gibco). Cells were monthly tested (negative) for mycoplasma contamination and maintained in a 37 °C humidity-controlled incubator with 5.0% CO_2_. Cell line rearrangements are as follows: Reh (TEL-AML1), THP-1 (MLL-AF9), MV4-11 (MLL-AF4), Nomo1 (MLL-AF9), OCI-AML2 (MLL-AF6), OCI-AML3 (none detected), and P31FUJ (MLLT10-PICALM). Reh, THP-1, MV4-11, Nomo1, OCI-AML2, OCI-AML3, and P31FUJ were obtained from the Cancer Cell Line Encyclopedia (https://portals.broadinstitute.org/ccle/home). HEK293FT cells (Invitrogen) were used at fewer than 30 passages. STR profiling was used to confirm all cell line identities upon arrival.

### Library and virus production

Pooled libraries for expression of dual sgRNAs were generated as detailed previously^8^ and cloned into pPapi (Addgene 96921). Briefly, 552 *S. aureus* oligonucleotides were mixed with 552 *S. pyogenes* oligonucleotides at 5 µM each for extension with NEBNext (New England Biolabs) with annealing at 48 °C. Purified dsDNA was purified and ligated using 100 cycles of Golden Gate assembly with 100 ng insert and 500 ng of vector using BsmBI and T7 ligase per reaction. Libraries were were isopropanol precipitated and transformed into STBL4 electrocompetent cells. The 300k library resulted in 304,704 total pairings whereby 286,225 of these perturbations were gene;gene KOs and 15,008 were gene;non-targeting KOs. At 24 h before transfection, a density of 18 × 10^6^ HEK293FT cells were seeded in each T175 flask in 30 ml of DMEM + 10% FBS. Transfection was performed using TransIT-LT1 (Mirus) transfection reagent. Beginning with 6 ml of Opti-MEM, a DNA mixture was prepared consisting of packaging plasmid pCMV_VSVG (Addgene 8454, 10 µg), psPAX2 (Addgene 12260, 40 µg), and the sgRNA-containing vector (e.g., pPapi, 40 µg). After a 20-min incubation the solution was added dropwise to the T175 flask and incubated for 6–8 h. Fresh media was added to the cells and collected 36 h later and snap frozen.

### Library screening

To determine lentiviral titer, cell lines were transduced in 12-well plates with 0, 20, 100, 200, 300, 500 µL virus with 3.0 × 10^6^ cells per well. After 8 h cells were pelleted by centrifugation, viral supernatant aspirated and passaged into a 6-well at 1.0 × 10^6^ cells per well. Two days post-transduction, cells were split into 2 6-wells and puromycin added to one of the wells. After 3 additional days, cells were counted for viability by trypan exclusion. A viral dose resulting in 30–50% transduction efficiency was used for subsequent library screening.

Primary pool transductions were performed with enough cells to achieve a representation of at least 1000 cells per sgRNA per replicate. For the 300k library screen, THP-1 and Reh cells were cultured in 3 L spinner flasks (Corning) in an incubator outfitted with magnetic stir bar base. We observed doubling times for Reh and THP-1 to be 30 h and 24 h respectively. For all screens, the cells were split at a density to maintain a representation of at least 1000 cells per sgRNA. For THP-1, MV4-11, Nomo1, P31FUJ, OCI-AML2, and OCI-AML3, cells were supplemented with 8 µg/ml polybrene. Reh cells were supplemented with 2 µg/ml polybrene. Selection was with 1 µg/ml puromycin for THP-1, Reh, and Nomo1 and 0.5 µg/ml puromycin for OCI-AML2, OCI-AML3, MV4-11, and P31FUJ. After 3 days, puromycin selection was maintained at half the initial dose for the remainder of the screen. At the final timepoint of 21 days, cells were pelleted by centrifugation, washed with PBS, and frozen promptly for genomic DNA isolation. All screens were run in duplicate.

### Genomic DNA preparation and sequencing

Genomic DNA was isolated using the QIAamp DNA Blood Maxi Kit (Qiagen). PCR, sequence adaptor barcoding, cleanup, sequencing, and data deconvolution were carried out as described^8^. At the PCR stage, pilot library plasmid DNA (pDNA) was diluted to 10 ng, while 300k library pDNA was diluted to 0.05 pg to closer match gDNA transcript levels. All PCR was carried out for 28 cycles.

### Data analysis

To score genetic interactions we used a custom python package, gnt (https://github.com/gpp-rnd/gnt), available on the python package index. We use log-fold changes (LFCs) as inputs to the scoring pipeline. We define ${y}_{{ij}}$ as the observed LFC of a sgRNA pair $i$, $j$, and $\hat{{y}_{{ij}}}$ as this pair’s expected LFC. We then calculate the residual ${y}_{{ij}}-\hat{{y}_{{ij}}}$ to generate an interaction score. To define expected LFCs, we fit a linear regression for each sgRNA, $i$, saying $\hat{{y}_{i}}={m}_{i}\cdot x+{b}_{i},$ where $i$ is the LFC of each sgRNA individually and ${m}_{i}$ and ${b}_{i}$ are the fit slope and intercept for sgRNA $i$. We refer to $i$ as the anchor sgRNA and its pairs as target sgRNA. We then z-score residuals within each anchor sgRNA, as done previously^39^.

To aggregate interaction scores at the gene level, we summed the z-scored residuals, ${z}_{{ij}}$, for all constructs $i$, ${j}$ targeting the gene pair $I$, $J$, fixing $I$ as the anchor gene, and dividing by the square root of the number of constructs targeting $I$, $J$. We repeated this calculation, fixing ${J}$ as the anchor gene. We summed scores for both orientations and divided by $\sqrt{2}$ to arrive at a gene-level z-score.

### Quantitative real time PCR

RNA was isolated with Qiagen RNeasy Plus Micro Kit (Qiagen 74134) according to the manufacturer’s protocol. cDNA synthesis was performed using the Superscript III First-Strand Synthesis System for RT-PCR (Invitrogen 18080-051) according to the manufacturer’s protocol. qPCR plates were prepared with Power SYBR Green PCR Master Mix (Applied Biosystems 4368706) with probes to ING1a (For-TCGGAGACAGTTTCAGGC, Rev-CGACTGAAGCGCTCGTA), ING1b (For- GGACTACCTGGACTCCAT, Rev-CGACTGAAGCGCTCGTA), ING1 exon2 (For-AGACCATGGACAAAGCCCTG, Rev- CCTTGCACCTCAACAAAGGC), ING2a (For- AGAGGAACGTGTCTGTGCTG, Rev- TGGAGAAGCTGCTGTAGACG), ING2b (For- CAGCATGTTTTGCGGTGATGT, Rev- GCTGTAGACGTTTCTTCTGGTTT), ING2 exon2 (For-TGCTGAAAGTGAACGAGCCT, Rev-ATCACGGCTTTCACTGGTCC), and B2M (For-ACTGAATTCACCCCCACTGA, Rev-CCTCCATGATGCTGCTTACA). B2M was used as a control across all samples tested. Samples were cycled and analyzed on a BioRad CFX Opus 384 Real-Time PCR System (BioRad).

### RNA sequencing sample preparation and analysis

THP-1, Reh and MV4-11 cells were transduced with mCherry *S. pyogenes* Cas9 and sgRNA delivery vector (pXPR_044, Broad GPP) and EGFP *S. pyogenes* Cas9 and sgRNA delivery vector (pAW12.lentiguide.GFP, Addgene 104374) carrying safe harbor, MTA1, MTA2 and CHD4 sgRNAs. Two sgRNAs were used for each gene to ensure complete KO, sequences found in Supplementary Data 2. Greater than 60% of cells expressed fluorescent markers, otherwise cells were sorted on a Sony SH800 sorter. Gating strategy can be found in Supplementary Fig. 10. RNA was isolated with Qiagen RNeasy Plus Micro kit according to the manufacturer’s protocol and ensuring RIN values greater than 7. Libraries were prepared first with Poly-A enrichment using magnetic oligo(dT)-beads (Invitrogen), then ligated to RNA adaptors for sequencing.

Data were aligned with STAR aligner^40^ to the hg38 genome assembly using the “Basic” two-pass mode. The option “--outFilterScoreMinOverLread“ was set to 0.2 to allow mapping of shorter fragments. Raw gene counts were obtained using STAR aligner (2.6.0c) by adding the option “--quantMode GeneCounts” to the mapping script. NCBI RefSeq GTF file was used for gene counting purposes. The merged length of all exons for each protein-coding gene was used to correct raw counts. TPM was calculated using the following formula in R: “tpm.mat < - t(t(x) * 1e6/colSums(x))” where x is the gene length-corrected expression matrix. Log_2_ fold-change was computed relative to control (safe harbor sgRNA) cells. For differential gene expression, the raw counts matrix was used. Genes with less than ten reads across all the libraries were removed. DESeq2 was used to identify differentially expressed genes with default settings^41^. Independent Hypothesis Weighting (IHW) was implemented to identify genes that are significantly differentially expressed^42^. In Fig. 3d heatmaps, differential cutoffs included TPM greater than 1, LFC greater than 2 or less than −2 and *p*-value less than 0.05.

R (version 3.5.1), Python (version 2.8) and Graphpad Prism (version 9) were used for visualization.

### ING5 Pfam targeting assay

THP-1 cells were transduced with mCherry *S. pyogenes* Cas9 and sgRNA vector (pXPR_044, Broad GPP) targeting ING5 regions and EGFP *S. pyogenes* Cas9 and sgRNA vector (pAW12.lentiguide.GFP) targeting ING4 or Safe Harbor. Sequences for sgRNAs can be found in Supplementary Data 2. Cells were collected after 6 or 14 days, suspended in PBS with 0.5% bovine serum albumin, and assayed by flow cytometry (Beckman Coulter CytoFLEX). Populations were analyzed with FlowJo (BD, version 10) by first gating for live populations with forward and side scatter (see Supplementary Fig. 10a and Supplementary Data 6). Untreated THP-1 cells were used to set the threshold gate at <1%.

### Reporting summary

Further information on research design is available in the Nature Portfolio Reporting Summary linked to this article.

## Supplementary information

Supplementary Information

Description of Additional Supplementary Files

Supplementary Data 1

Supplementary Data 2

Supplementary Data 3

Supplementary Data 4

Supplementary Data 5

Supplementary Data 6

Reporting Summary

## Data Availability

The raw RNA-seq and CRISPR screening data generated in this study have been deposited in the Gene Expression Omnibus (GEO) and are available without restriction under accession number GSE215348. Processed CRISPR screening and RNA-seq data are available in Supplementary Data 3 and 5. The DepMap publicly available data used in this study are available in the online database https://depmap.org/portal/download/all/^18^ with DepMap release version found in respective figure legend. Source data are provided with this paper.

## Source data

Source Data

## Code availability

All customized code used for analysis and notebooks is available on GitHub: https://github.com/gpp-rnd/gnt.
