# Supplementary material for: Chromatin complex dependencies reveal targeting opportunities in leukemia
Source: Nat Commun. 2023 Jan 27;14:448. doi: 10.1038/s41467-023-36150-7 (PMC9883437; doi:10.1038/s41467-023-36150-7)
Supplement: Supplementary file 1 — Supplementary Information [file 41467_2023_36150_MOESM1_ESM.pdf]

Supplementary Information

Chromatin complex dependencies reveal targeting opportunities in leukemia

Supplementary Figures

Supplementary Figure 1. Characterization of 268 chromatin regulator genes selected for screening

Supplementary Figure 2. Cas9 ortholog and replicate performance comparison for the pilot library screen

Supplementary Figure 3. Pilot library screen data

Supplementary Figure 4. Cas9 ortholog and replicate performance comparison for the 300k library screen

Supplementary Figure 5. Single gene knockout data from the 300k library screen

Supplementary Figure 6. ING1 and ING2 expression in response to knockout

Supplementary Figure 7. Validation screen performance

Supplementary Figure 8. Remodeling and repressive complex dependencies

Supplementary Figure 9. Gene expression in response to NuRD member knockout

Supplementary Figure 10. Flow cytometry and gating strategy

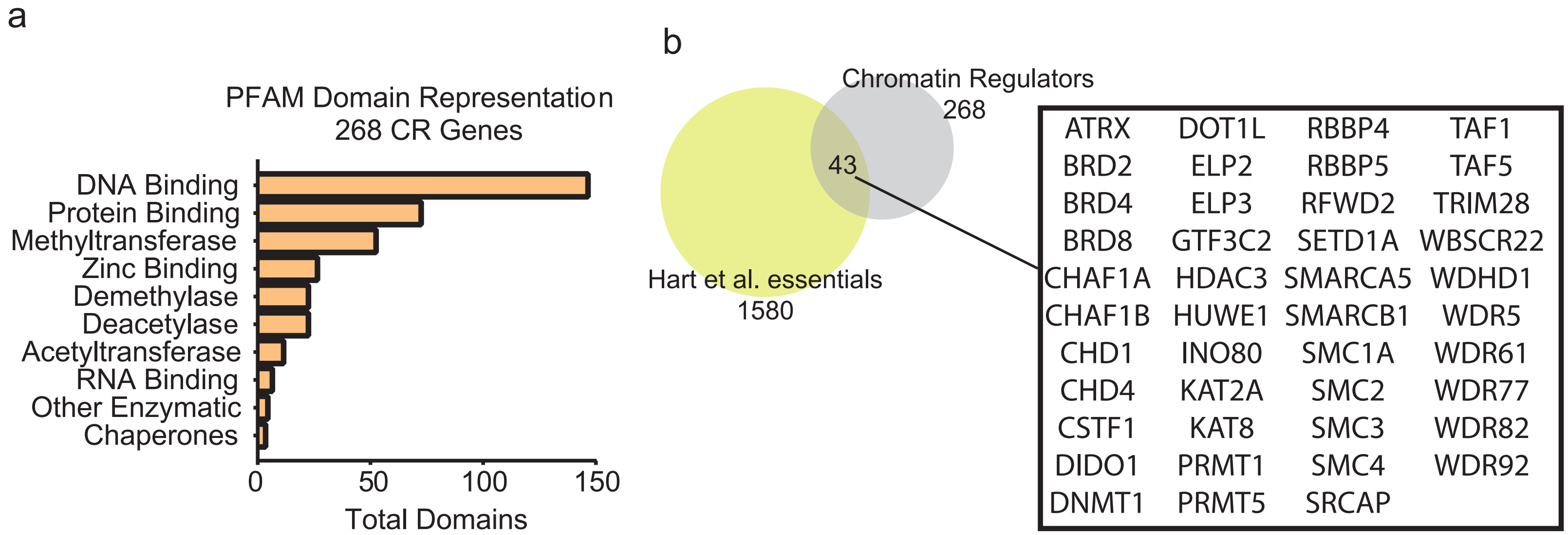

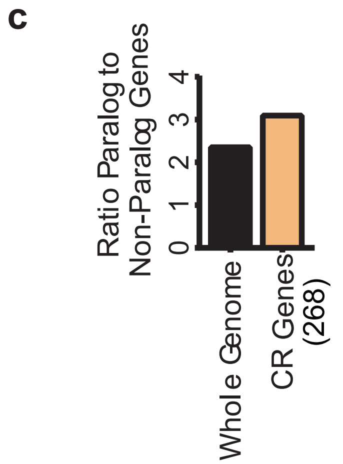

Supplementary Figure 1. Characterization of 268 chromatin regulator genes selected for screening. **a**, Representation of 50 PFAM domains across 268 chromatin regulator (CR) genes selected in this study. **b**, Venn diagram of essential genes reported in Hart et al. 2015 and the 268 selected chromatin regulators with the overlapping 43 genes labeled. **c**, Paralog ratio representation for chromatin regulator genes and whole genome (19,430 genes)[1].

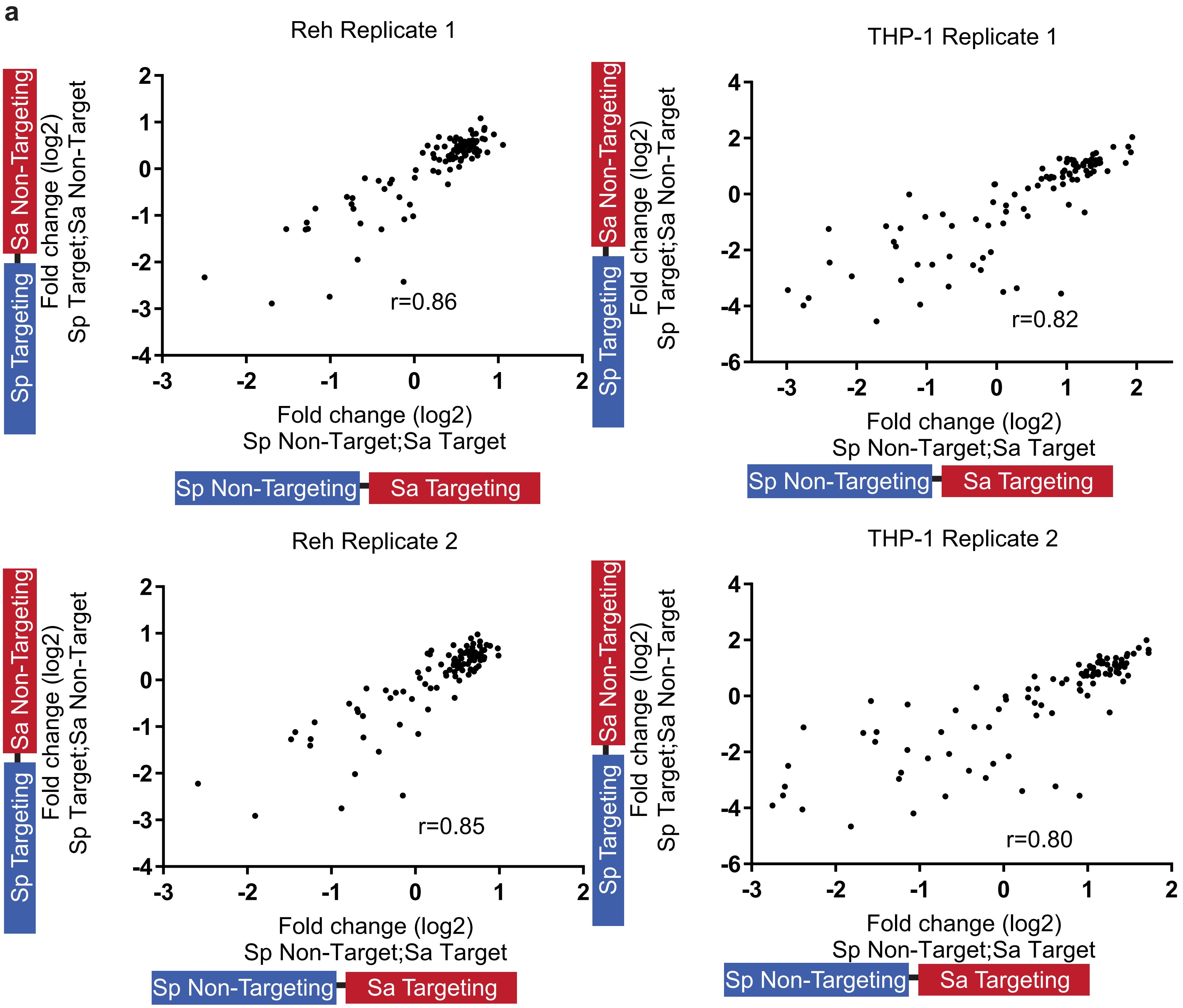

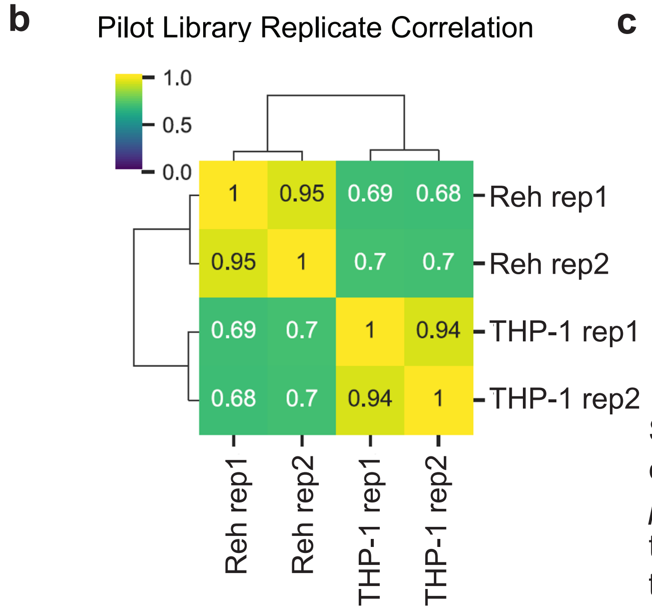

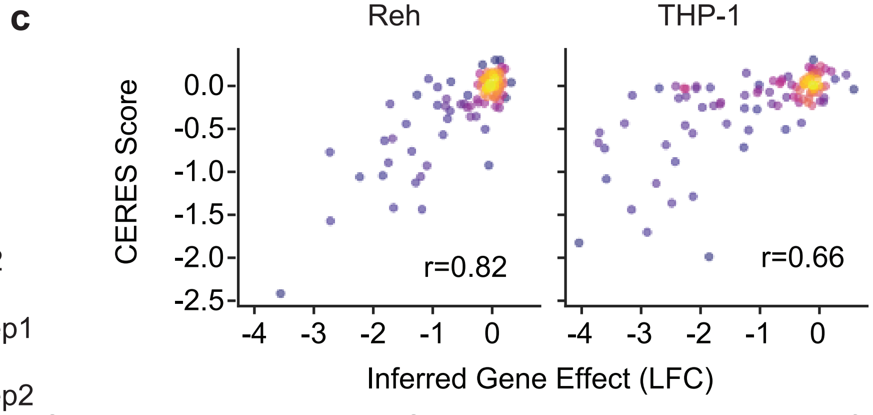

Supplementary Figure 2. Cas9 ortholog and replicate performance comparison for the pilot library screen. **a**, *S. aureus* (Sa) and *S. pyogenes* (Sp) Cas9 ortholog correlation when a single gene is targeted. **b**, Replicate correlation between THP-1 and Reh cell lines in the pilot library screens. **c**, Correlation between Reh and THP-1 40k library single knockout data with published DepMap[2] CERES scores. DepMap version 20q2 and correlations are pearson for all plots.

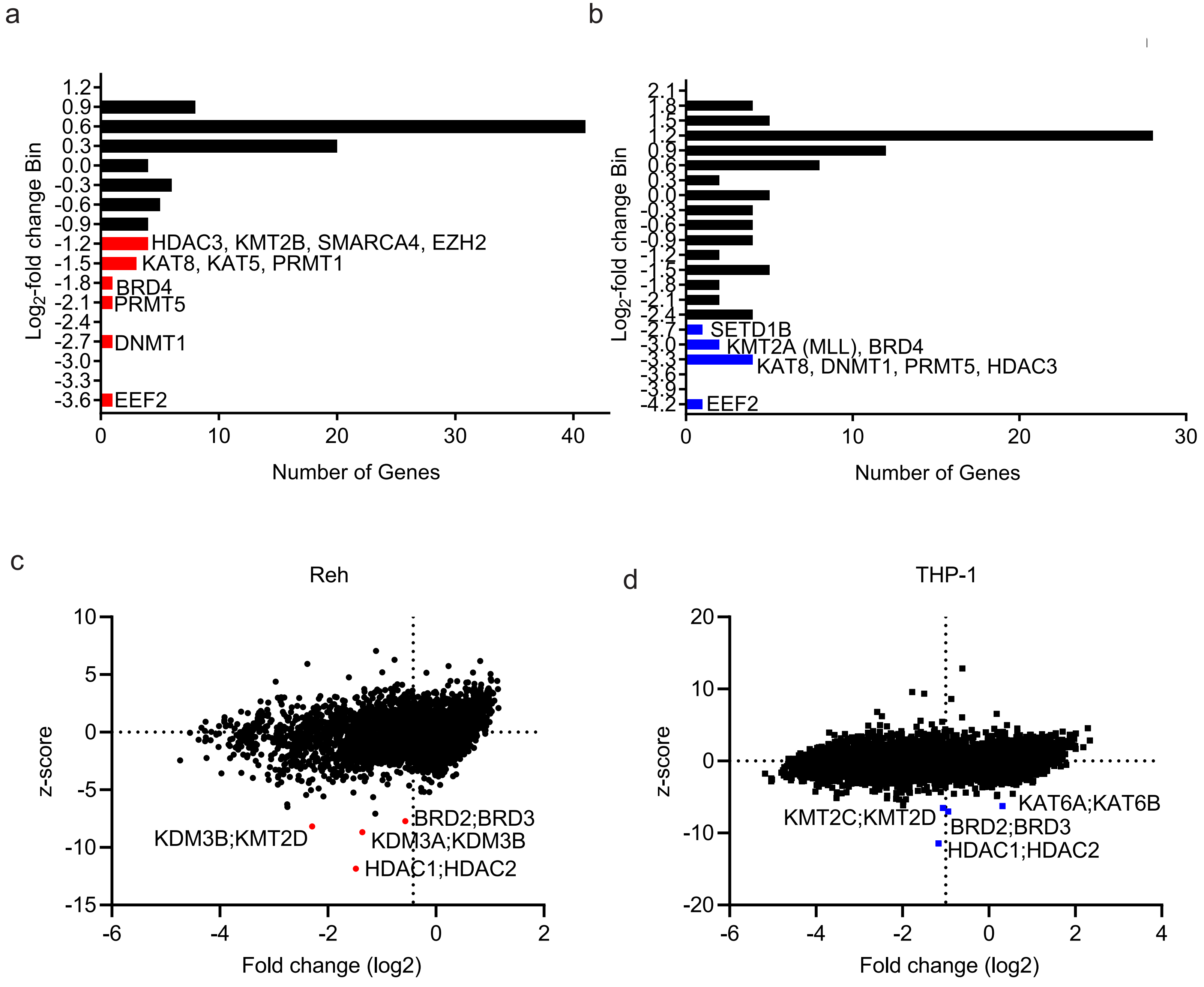

Supplementary Figure 3. Pilot library screen data. **a**, **b**, Single knockout data for Reh in **a** (top hits in red) and THP-1 in **b** (top hits in blue). EEF2 is an essential gene serving as a control. **c**, **d** Volcano plots of combinatorial knockout data (n=4743 gene pairs) depicting top scoring hits in Reh in **c** (red circles) and THP-1 in **d** (blue squares). Dotted lines placed at the mean values for each axis.

a

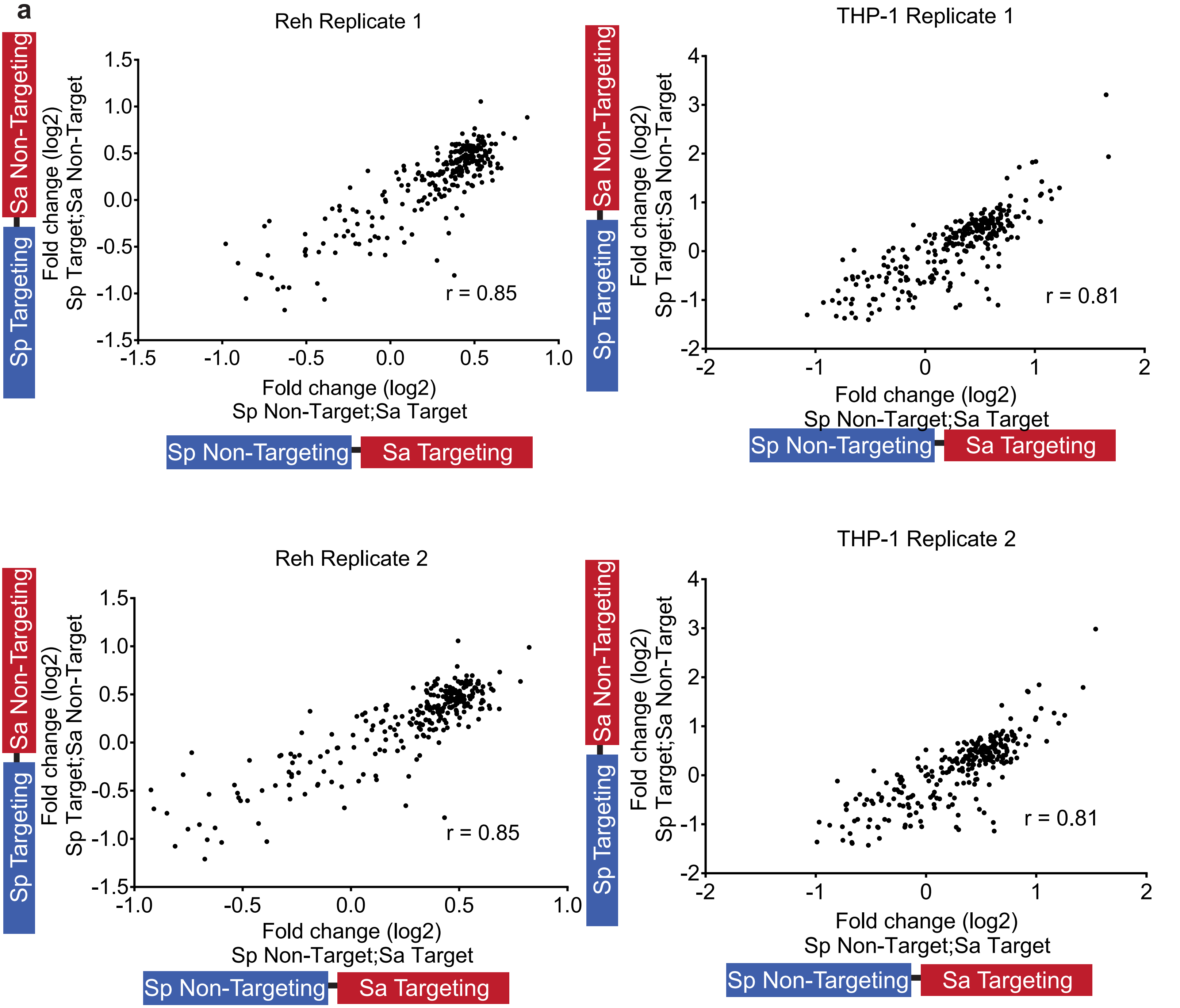

b

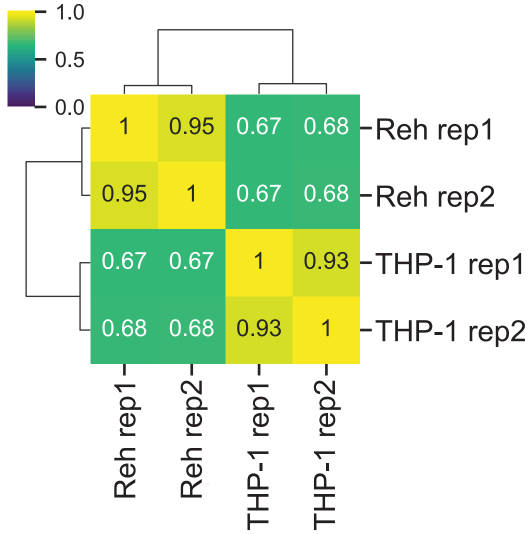

Supplementary Figure 4. Cas9 ortholog and replicate performance comparison for the 300k library screen. **a** S aureus (Sa) targeting guides are plotted on the x-axis and S pyogenes (Sp) targeting guides plotted on the y-axis. Guide data were averaged to collapse down by gene. Replicates for both Reh and THP-1 are displayed. **b** Replicate correlation between THP-1 and Reh cell lines in the 300k library screens. Correlations are pearson for all plots.

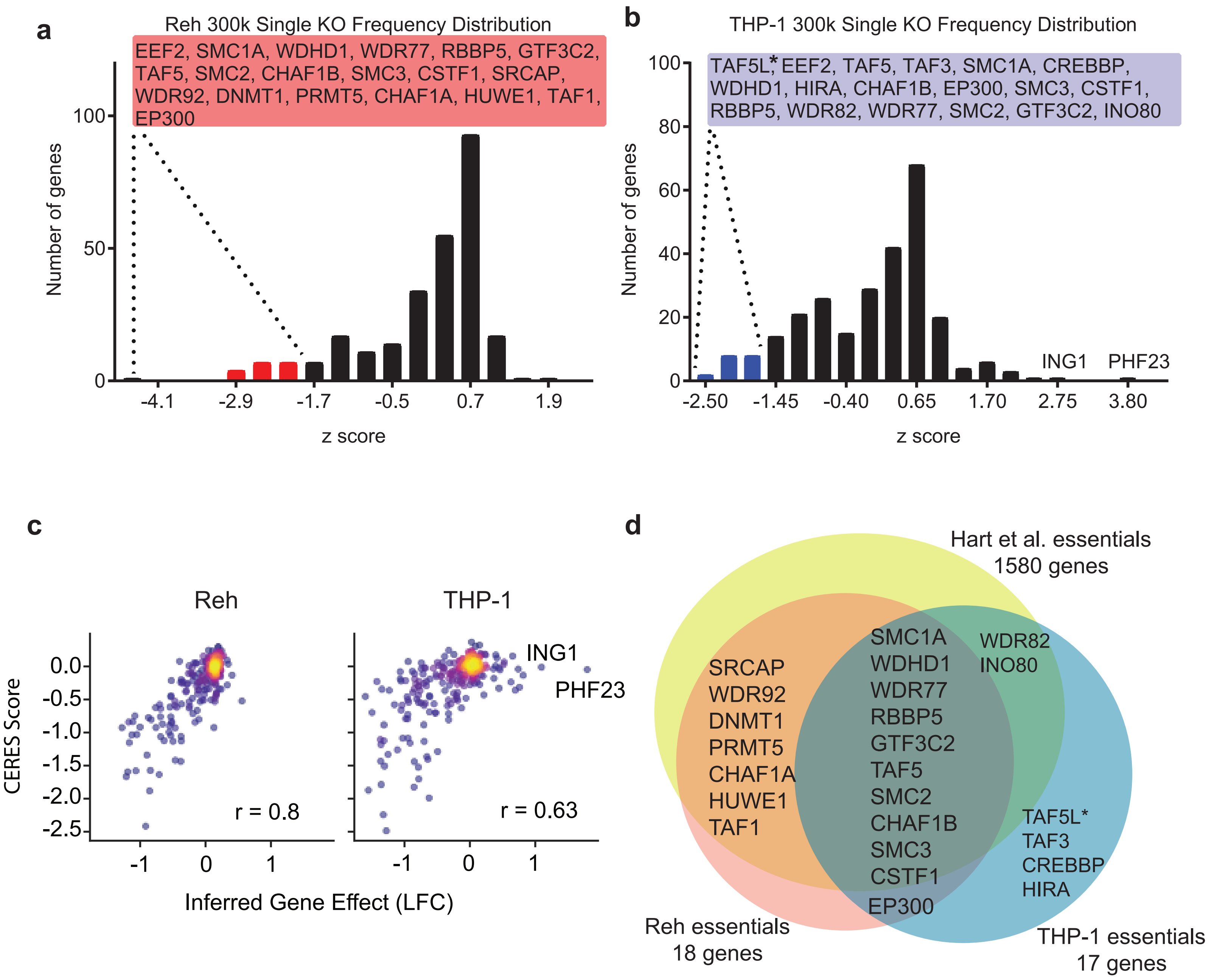

Supplementary Figure 5. Single gene knockout data from the 300k library screen. **a b**, Histograms of average single gene knockout (KO) representation for Reh in **a** and THP-1 in **b**. Genes were labeled essential based on 2 standard deviations below mean. EEF2 included as a positive control. THP-1 screen revealed ING1 and PHF23 genes as highly represented knockouts. **c**, Single knockout screening data compared to CERES score from Depmap[2] 20q2. Correlations are pearson. ING1 and PHF23 led to proliferation in the 300k library screen and are labeled on the plot. **d**, Venn diagram depicting intersection of essential single knockouts from both screens and prior published 1580 core essential genes[3]. TAF5L* is copy number amplified in THP-1 cell line and may score as a result of the DNA damage response.

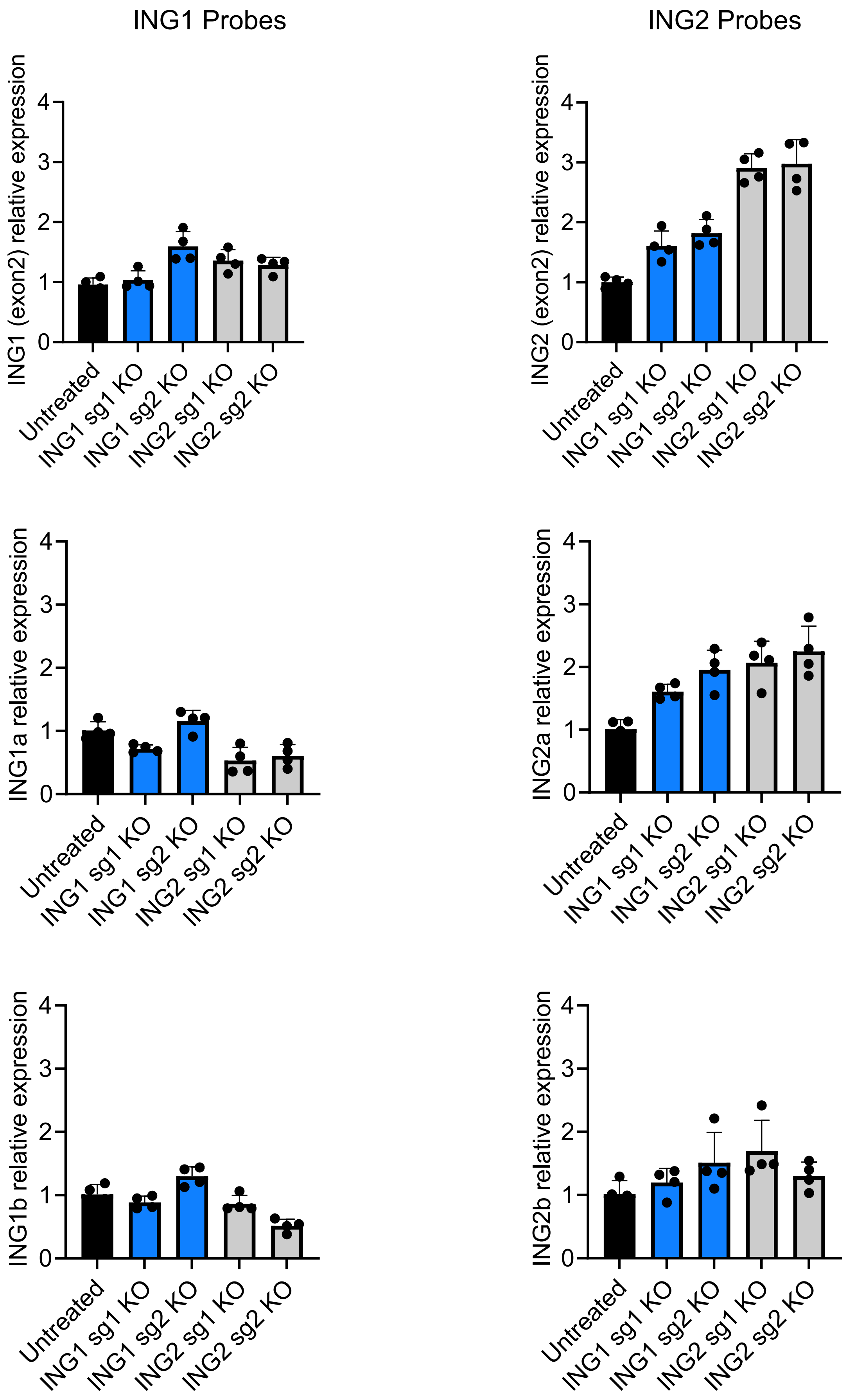

Supplementary Figure 6. ING1 and ING2 expression in response to knockout. THP-1 cells were treated with ING1 (blue) or ING2 (grey) sgRNAs (2 each gene, sg1 and sg2, see Supplementary Table 2 for sequences) and analyzed by quantitative real time PCR (QPCR) for expression of ING1 and ING2 by 3 probesets each. QPCR probe sequences targeted isoform a, isoform b, or exon 2 for each gene (see Methods for sequences). Quadruplicate data (n=4) are normalized to untreated cells, mean +/- standard deviation.

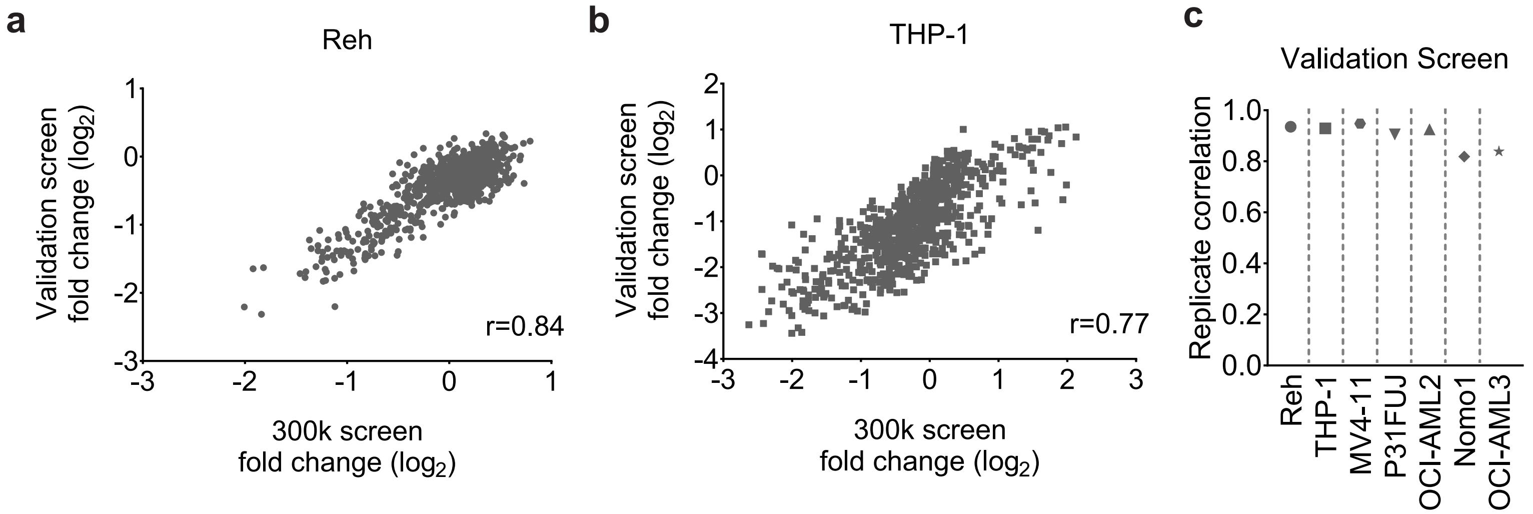

Supplementary Figure 7. Validation screen performance. **a b**, Scatter plots depicting the average log2-fold change between 741 overlapping gene-gene combinations in the 300k library scale screen and the validation screen for Reh in **a** and THP-1 in **b**. Pearson correlation depicted on plots. **c** Pearson coefficients between duplicate screens for each cell line screened.

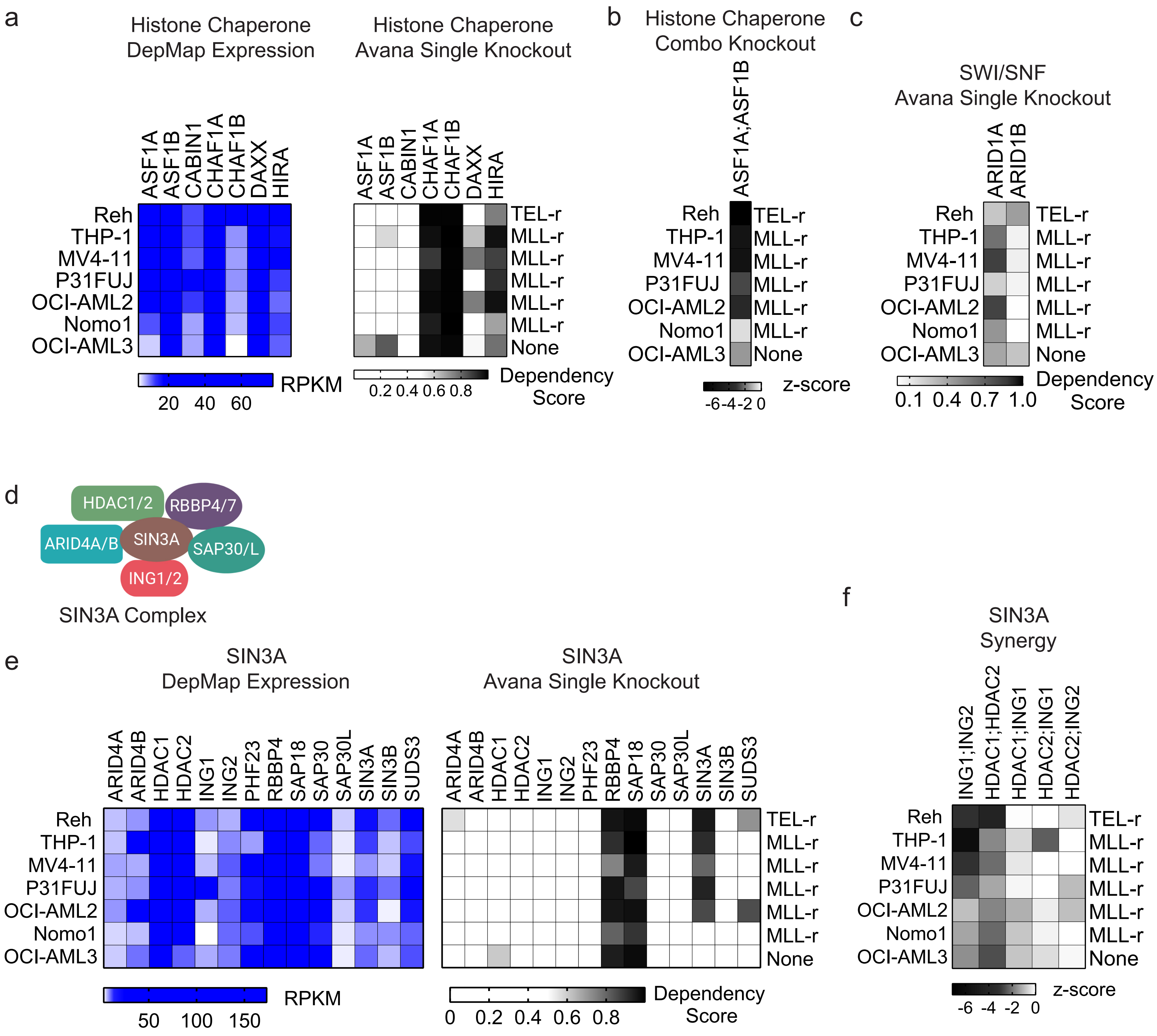

Supplementary Figure 8. Remodeling and repressive complex dependencies. **a**, Expression and single knockout data for 7 histone chaperones. **b**, ASF1A and ASF1B combinatorial knockout data from the validation screen. Data are from duplicate experiments (n=2, 4 distinct sgRNA pairs tested). **c**, ARID1A and ARID1B single knockout. **d**, SIN3A complex schematic. **e**, Expressioin and single knockout data for SIN3A complex proteins. **f** Combinatorial knockout data for SIN3A members from the validation library screen. Data are from duplicate experiments (n=2, 4 distinct sgRNA pairs tested). Data in **a**, **c** and **e** are from DepMap[2] 19q1.

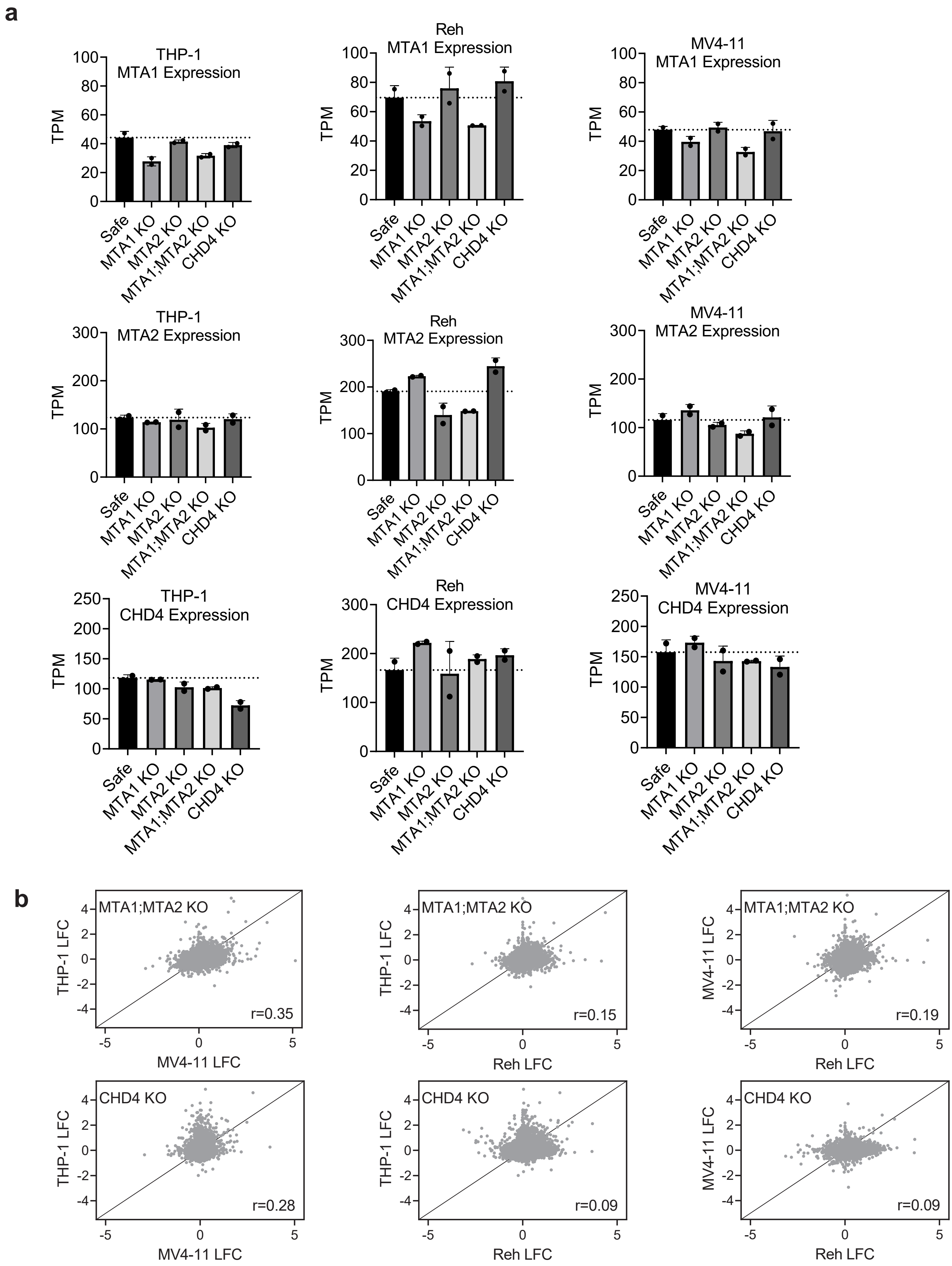

Supplementary Figure 9. Gene expression in response to NuRD member knockout. Figure legend on next page.

c

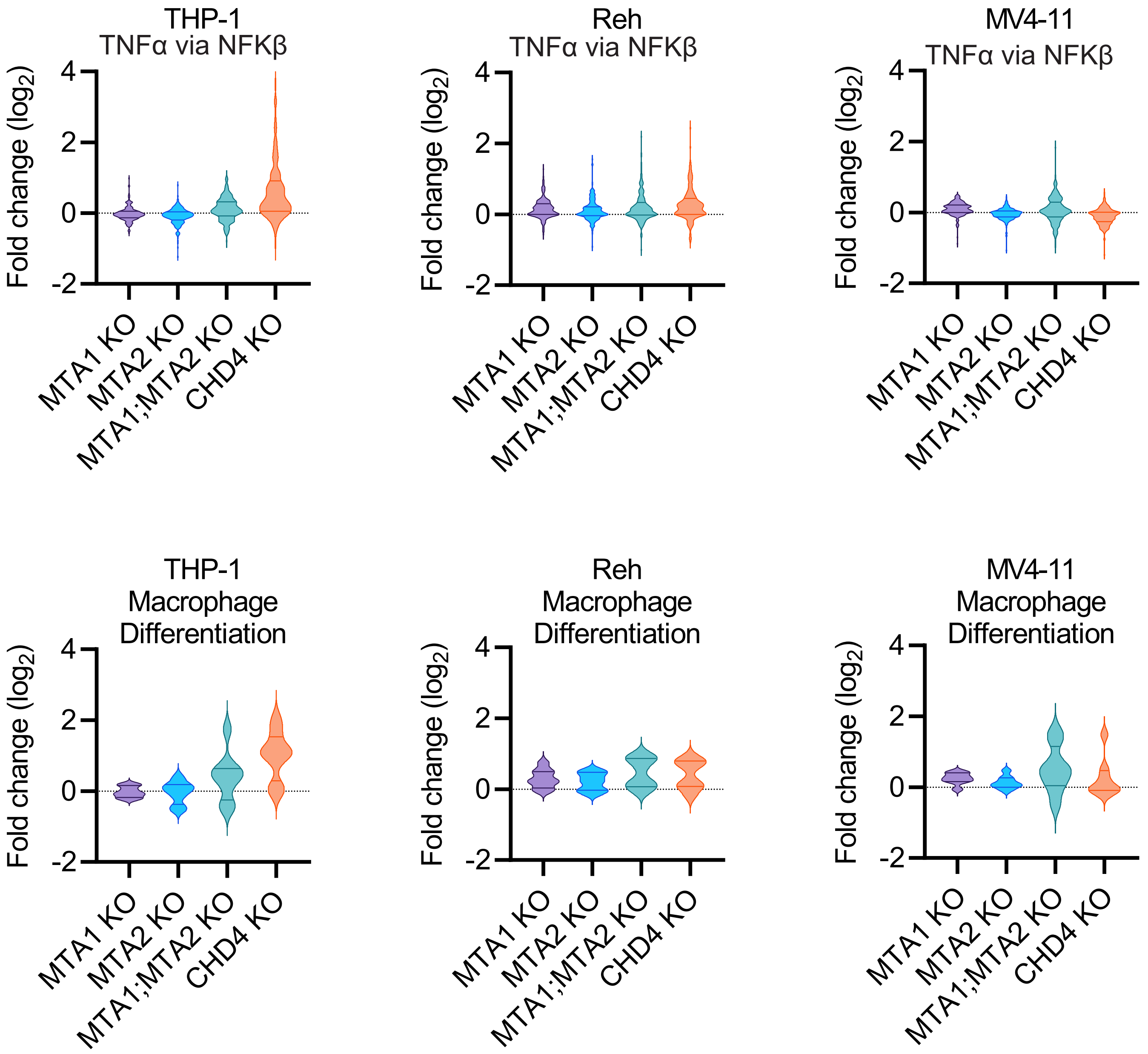

Supplementary Figure 9. Gene expression in response to NuRD member knockout. (continued) THP-1, Reh and MV4-11 cells were treated with Safe Harbor (Safe), CHD4, MTA1 and/or MTA2 sgRNAs (2 sgRNAs each gene, see Supplementary Table 2 for sequences) for 7 days and analyzed by RNAseq. **a**, MTA1, MTA2 or CHD4 expression (TPM) for the indicated knockout conditions. Dotted line at the Safe average. Duplicate data (n=2) are mean with standard deviation. **b**, All expression data (log_2 fold-change relative to safe) plotted for the indicated cell line comparisons. Correlations are pearson. **c**, Gene expression (log_2 fold-change relative to safe) for selected sets of Hallmark TNFa signalling via NFKB members (200 genes) and macrophage differenation markers (CD14, ITGAM, ITGAM1, APOE, CSF1, CSF1R, CD36, SPP1). Violin plots depict top and bottom quartiles with a solid line. All expression data in this figure are from two replicates.

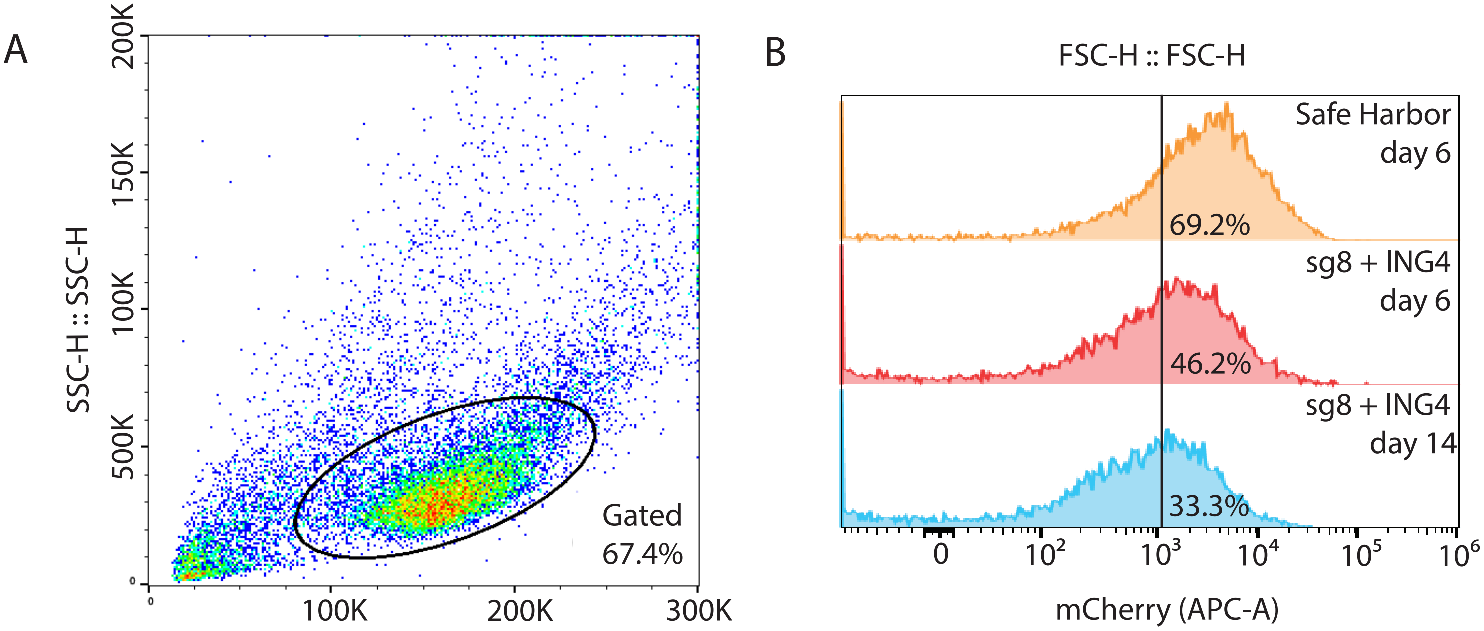

Supplementary Figure 10. Flow cytometry and gating strategy. **a**, Example plot of forward-scatter (FSC) and side-scatter (SSC) selection of live cells utilized for all samples tested. **b**, Histogram of replicate 1 timecourse of sg8 treated THP-1 cells. Percentage is mCherry positives with a gate set with untreated cells at <1% fluorescent positive.

Supplementary References

1. Ibn-Salem, J., Muro, E. M. & Andrade-Navarro, M. A. Co-regulation of paralog genes in the three-dimensional chromatin architecture. *Nucleic Acids Res.* **45**, 81–91 (2017).
2. Tsherniak, A. *et al.* Defining a cancer dependency map. *Cell* **170**, 564-576.e16 (2017).
3. Hart, T. *et al.* High-resolution CRISPR screens reveal fitness genes and genotype-specific cancer liabilities. *Cell* **163**, 1515–1526 (2015).
