# Supplementary material for: Chromatin complex dependencies reveal targeting opportunities in leukemia
Source: Nat Commun. 2023 Jan 27;14:448. doi: 10.1038/s41467-023-36150-7 (PMC9883437; doi:10.1038/s41467-023-36150-7)
Supplement: Supplementary file 2 — Description of Additional Supplementary Files [file 41467_2023_36150_MOESM2_ESM.pdf]

Description of Additional Supplementary Files

Chromatin complex dependencies reveal targeting opportunities in leukemia

Supplementary Data 1. Pfam domain representation among the 268 chromatin regulator genes screened in this study.

Supplementary Data 2. S pyogenes and S aureus CRISPR sgRNA sequences used in both pooled combinatorial libraries and follow-up experiments.

Supplementary Data 3. Combinatorial CRISPR screening data for the 300k, 40k and validation library screens.

Supplementary Data 4. Annotated mutations of the 268 chromatin regulator genes in the 7 leukemia cell lines tested (DepMap 22Q4).

Supplementary Data 5. RNA-seq expression data in THP-1, Reh and MV4-11 cells treated with CRISPR knockouts of NuRD complex members MTA1, MTA2, MTA1;MTA2, and CHD4 or Safe Harbor sgRNAs.

Supplementary Data 6. Percentage and number of fluorescent positive cells for each ING5, ING4 and Safe Harbor sgRNA tested at day 6 and 14 timepoints. A minimum of 10,000 gated (presumed live) cells were analysed. In two vector experiments, cells were also gated for GFP expression. See Supplementary Figure 10 for fluorescent gating strategy.
