# Supplementary material for: Chromatin complex dependencies reveal targeting opportunities in leukemia
Source: Nat Commun. 2023 Jan 27;14:448. doi: 10.1038/s41467-023-36150-7 (PMC9883437; doi:10.1038/s41467-023-36150-7)
Supplement: Supplementary file 9 — Reporting Summary [file 41467_2023_36150_MOESM9_ESM.pdf]

nature research

Corresponding author(s): Bradley Bernstein

Last updated by author(s): Jan 6, 2022

Reporting Summary

Nature Research wishes to improve the reproducibility of the work that we publish. This form provides structure for consistency and transparency in reporting. For further information on Nature Research policies, see our Editorial Policies and the Editorial Policy Checklist.

Statistics

For all statistical analyses, confirm that the following items are present in the figure legend, table legend, main text, or Methods section.

n/a	Confirmed	
☐	☒	The exact sample size (*n*) for each experimental group/condition, given as a discrete number and unit of measurement
☐	☒	A statement on whether measurements were taken from distinct samples or whether the same sample was measured repeatedly
☐	☒	The statistical test(s) used AND whether they are one- or two-sided<br>*Only common tests should be described solely by name; describe more complex techniques in the Methods section.*
☒	☐	A description of all covariates tested
☒	☐	A description of any assumptions or corrections, such as tests of normality and adjustment for multiple comparisons
☐	☒	A full description of the statistical parameters including central tendency (e.g. means) or other basic estimates (e.g. regression coefficient) AND variation (e.g. standard deviation) or associated estimates of uncertainty (e.g. confidence intervals)
☐	☒	For null hypothesis testing, the test statistic (e.g. *F*, *t*, *r*) with confidence intervals, effect sizes, degrees of freedom and *P* value noted<br>*Give P values as exact values whenever suitable.*
☒	☐	For Bayesian analysis, information on the choice of priors and Markov chain Monte Carlo settings
☒	☐	For hierarchical and complex designs, identification of the appropriate level for tests and full reporting of outcomes
☐	☒	Estimates of effect sizes (e.g. Cohen's *d*, Pearson's *r*), indicating how they were calculated

Our web collection on statistics for biologists contains articles on many of the points above.

Software and code

Policy information about availability of computer code

Data collection	No specialized software or code was used for data collection.
Data analysis	All customized code used for analysis and notebooks is available on GitHub: https://github.com/gpp-rnd/gnt<br><br>R (version 3.5.1), Python (version 2.8) and Graphpad Prism (version 9) were used for visualization.<br><br>FlowJo (version 10) was used to process flow data and contour plots.<br><br>STAR Aligner (2.6.0c) was used to process RNA-seq reads.

For manuscripts utilizing custom algorithms or software that are central to the research but not yet described in published literature, software must be made available to editors and reviewers. We strongly encourage code deposition in a community repository (e.g. GitHub). See the Nature Research guidelines for submitting code & software for further information.

Data

Policy information about availability of data

All manuscripts must include a data availability statement. This statement should provide the following information, where applicable:

- Accession codes, unique identifiers, or web links for publicly available datasets
- A list of figures that have associated raw data
- A description of any restrictions on data availability

The raw RNA-seq and CRISPR screening data generated in this study have been deposited in the Gene Expression Omnibus (GEO) and are available without restriction under accession number GSE215348. https://www.ncbi.nlm.nih.gov/geo/query/acc.cgi?acc=GSE215348 Processed CRISPR screening and RNA-seq data

are available in Supplementary Data 3 and 5. The DepMap publicly available data used in this study are available in the online database https://depmap.org/portal/download/all/ ref18 with DepMap release version found in respective figure legend. Source data are provided with this paper.

Field-specific reporting

Please select the one below that is the best fit for your research. If you are not sure, read the appropriate sections before making your selection.

☒ Life sciences ☐ Behavioural & social sciences ☐ Ecological, evolutionary & environmental sciences

For a reference copy of the document with all sections, see nature.com/documents/nr-reporting-summary-flat.pdf

Life sciences study design

All studies must disclose on these points even when the disclosure is negative.

Sample size	All pooled screens were performed in duplicate, as is standard in the field. Each tested gene is targeted by 4 separate sgRNAs increasing confidence. Strong correlation between duplicates in this format suggests an adequate sample size.
Data exclusions	No data were excluded.
Replication	All CRISPR screens and RNA-seq performed in duplicate. For other experiments, replicates and sgRNAs are indicated on figure legends and displayed on plots when n<10.
Randomization	Experiments were carried out on low passage cell lines that were grown as bulk populations. Randomization not relevant here.
Blinding	Blinding was not relevant, since there were no expected outcomes or predefined outcome measures.

Reporting for specific materials, systems and methods

We require information from authors about some types of materials, experimental systems and methods used in many studies. Here, indicate whether each material, system or method listed is relevant to your study. If you are not sure if a list item applies to your research, read the appropriate section before selecting a response.

Materials & experimental systems

n/a	Involved in the study	
☒	☐	Antibodies
☐	☒	Eukaryotic cell lines
☒	☐	Palaeontology and archaeology
☒	☐	Animals and other organisms
☒	☐	Human research participants
☒	☐	Clinical data
☒	☐	Dual use research of concern

Methods

n/a	Involved in the study	
☒	☐	ChIP-seq
☐	☒	Flow cytometry
☒	☐	MRI-based neuroimaging

Eukaryotic cell lines

Policy information about cell lines

Cell line source(s)	HEK293FT (Invitrogen). THP-1, Reh, OCI-AML2, OCI-AML3, Nomo1, P31FUJ, MV4-11 (Cancer Cell Line Encyclopedia, https://portals.broadinstitute.org/ccle/home).
Authentication	STR profiling (ATCC).
Mycoplasma contamination	All cell lines were tested monthly for mycoplasma contamination and all were negative.
Commonly misidentified lines (See ICLAC register)	None.

Flow Cytometry

Plots

Confirm that:

☒ The axis labels state the marker and fluorochrome used (e.g. CD4-FITC).

☒ The axis scales are clearly visible. Include numbers along axes only for bottom left plot of group (a 'group' is an analysis of identical markers).

☒ All plots are contour plots with outliers or pseudocolor plots.

☒ A numerical value for number of cells or percentage (with statistics) is provided.

Methodology

Sample preparation	Cells were collected and resuspended in PBS with 0.5% bovine serum albumin.
Instrument	Beckman Coulter Cytoflex or Sony SH800 sorter
Software	FlowJo (version 10) was used to analyze datasets.
Cell population abundance	At least 10,000 live cells were analyzed in all experiments.
Gating strategy	Live cells were gated based on forward and side scatter. Example in Extended Data Fig. 10. mCherry and GFP thresholds were set using THP-1, MV4-11, and Reh cells without the fluorescent genes and setting this at <1%.

☒ Tick this box to confirm that a figure exemplifying the gating strategy is provided in the Supplementary Information.
